# Taxonomic Richness of Yeasts in Japan within Subtropical and Cool Temperate Areas

**DOI:** 10.1371/journal.pone.0050784

**Published:** 2012-11-30

**Authors:** Masako Takashima, Takashi Sugita, Bui Hong Van, Megumi Nakamura, Rikiya Endoh, Moriya Ohkuma

**Affiliations:** 1 Microbe Division/Japan Collection of Microorganisms, RIKEN BioResource Center, Wako, Saitama, Japan; 2 Department of Microbiology, Meiji Pharmaceutical University, Tokyo, Japan; New York State Health Department and University at Albany, United States of America

## Abstract

**Background:**

An understanding of the role of yeasts in the environment has been uncertain because estimates of population size and diversity have often been based on species identifications that were determined from a limited number of phenotypic characteristics. DNA-based species identification has now become widely used, allowing an accurate assessment of species in different habitats. However, there are still problems in classification because some genera are polyphyletic. Consequently, the identification of yeasts and measurement of their diversity at the genus level remains difficult, as does assignment of genera to higher taxonomic ranks.

**Methodology/Principal Findings:**

A total of 1021 yeast strains was isolated from soil samples and plant materials collected from Japan’s subtropical Iriomote Island and the cool temperate Rishiri Island. Based on sequence analyses of the D1/D2 domain of the LSU rRNA gene, these 1021 strains were tentatively classified into 183 species, with apparent new species accounting for approximately half of the total species isolated (60 and 46, Iriomote and Rishiri, respectively). The yeast species composition was statistically different between the two sites with only 15 species in common. Rarefaction curves of respective sources/areas gave distinctive patterns when the threshold of sequence identity became broader, indicating that the yeast diversity was distinct at the different taxonomic levels compared.

**Conclusions/Significance:**

Our isolation study of yeasts in Japan has enabled us to expand the inventory of species diversity because a large number of new species was observed in the sampling areas. Further, we propose use of a particular diversity threshold as an “indicator” to recognize species, genera and higher taxonomic ranks.

## Introduction

Since the Convention on Biological Diversity went into effect in 1993, studies of species diversity have increased dramatically and are aimed at conserving biodiversity and ensuring sustainable use. This has resulted in around 500–600 prokaryote species now being described each year (http://www.bacterio.cict.fr/). Unfortunately, this statistic has not been published for yeasts; 56 new species were listed in 2010 in MycoBank (http://www.mycobank.org/), suggesting that many yet undescribed yeast species exist.

Yeasts are phylogenetically divided into two groups: ascomycetous and basidiomycetous. Most ascomycetous yeasts are members of the Saccharomycotina [1], and they may not be as common as basidiomycetous yeasts in certain environments [2]. In contrast, basidiomycetous yeasts appear to be a much larger group and are phylogenetically distributed in every lineage of the Basidiomycota: Agaricomycotina, Pucciniomycotina and Ustilaginomycotina [1]. In phylogenetic trees, some clades consist solely of yeast species, while in other clades yeast species are intermingled with filamentous fungi.

According to present isolation data, basidiomycetous yeast genera, such as the genera *Cryptococcus* and *Sporobolomyces,* are common in soils and on plant leaves. However, because studies often relied on limited phenotypic data for identification [3], [4], it is likely that many of the species were misidentified, thus obscuring species relationships and knowledge of their ecology. The recent application of gene sequence analysis for species identification has brought new perspective to understanding the diversity and ecology of yeasts [5]. Many previous studies have focused on understanding species boundaries, but less emphasis was placed on recognizing genera and families. Because many genera are polyphyletic [1], it is difficult to know the extent of yeast diversity and to estimate its role on form and function in nature [6].

Japan consists of four large islands and many others that form a line from northeast to southwest, approximately 45°N148°E to 24°N122°E. Since the northern part belongs to the subarctic zone and the southern part to the subtropical zone, the two areas were expected to provide contrasting diversity. No comprehensive database of yeast species has been established for Japan. The present study was undertaken to determine the species diversity of yeasts in Japan, and then to further understand species diversity by comparing results from our study with similar studies from the USA, European and other Asian countries.

## Results and Discussion

### Isolation Results

A total of 1021 strains (443 from Iriomote Island and 578 from Rishiri Island) were isolated and classified into 183 species (105 and 93, respectively) based on the D1/D2 sequence similarity with the type strain of described species according to Kurtzman & Robnett [7] and Scorzetti et al. [8] (Table 1). Of these, 104 appeared to be new species; the ratio of new species (number of new candidates/number of identified) being 57% and 49% for Iriomote and Rishiri, respectively. Since 1312 species are listed in “The Yeasts, A Taxonomic Study” 5^th^ edition, which is a cardinal book on yeast systematics published in 2011 [1], the number of species identified thus corresponds to 14% of those listed, around 8% being new species candidates.

**Table 1:** Summary of yeast strains on plants and in soil samples from Rishiri Island and Iriomote Island.

	Iriomote Island							
	Plant sample				Soil sample			
	known species	number of strains	new species candidates	number of strains	known species	number of strains	new species candidates	number of strains
Agaricomycotina	11	60	21	54	8	23	16	93
Pucciniomycotina	12	42	12	30	3	10	2	2
Ustilaginomycotina	5	41	8	21	1	4	1	1
Saccharomycotina	3	10	3	6	9	33	5	13
Subtotal	31	153	44	111	21	70	24	109
Total*	105 species 443 strains (60 new species candidates)							
	**Rishiri Island**							
	**Plant sample**				**Soil sample**			
	**known species**	**number of strains**	**new species candidates**	**number of strains**	**known species**	**number of strains**	**new species candidates**	**number of strains**
Agaricomycotina	11	59	4	6	12	109	13	45
Pucciniomycotina	8	195	16	48	4	12	7	15
Ustilaginomycotina	4	12	2	2	1	2	0	0
Saccharomycotina	2	4	2	3	8	43	5	23
Subtotal	25	270	24	59	25	166	25	83
Total*	93 species 578 strains (46 new species candidates)							

* number of unique species.

15 species were commonly isolated from both sampling areas, as given in the text.

**Figure 1:**
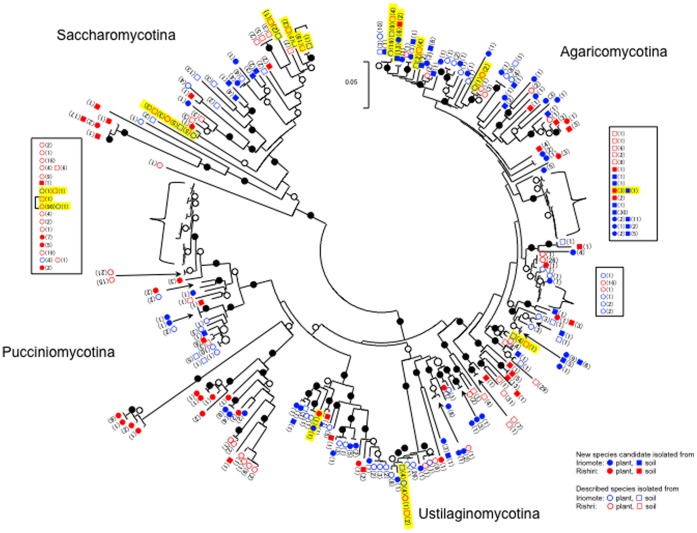
A neighbor-joining tree of isolates based on the D1/2 region unique sequences. The evolutionary distance (refer to the bar) was calculated according to Kimura [26]. Bootstrap values [27] were summarized by black dots (indicating at least 90% support) or by black rings (indicating at least 60% support). Blue solid ring, new species candidate isolated from a plant of Iriomote Island; blue open ring, described species isolated from a plant of Iriomote; blue solid square, new species candidate isolated from soil of Iriomote; blue open square, described species isolated from soil of Iriomote; blue solid ring, new species candidate isolated from a plant of Rishiri Island; red open ring, described species isolated from a plant of Rishiri; red solid square, new species candidate isolated from soil of Rishiri; red open square, described species isolated from soil of Rishiri. Numerals in brackets after ring or square indicate the number of isolates. Yellow color on OTUs indicates this species was isolated from both islands.

A neighbor-joining tree of isolates based on the unique sequences (D1/D2 region of the LSU rRNA gene) showed that the species were widely distributed on the phylogenetic tree, and we found no branch particular to the sampling area or source (Figure 1 and Figure S1 (a)–(d)). Furthermore, one third of new species candidates (39 species, indicated by “asterisk” in Figure S1 (a)-(d).) showed less than 97% sequence similarity to the phylogenetically closest described species, indicating that new species candidates obtained in this study will contribute to filling in missing areas of the fungal tree of life.

As shown in Table 1, more than 86% of the isolates were basidiomycetous yeasts and less than 14% were ascomycetous. In the former, strains in Agaricomycotina were most frequently isolated from Iriomote samples whereas strains in Pucciniomycotina and Agariomycotina were frequently isolated in about the same quantity from Rishiri Island. Basidiomycetous yeasts were isolated more frequently than ascomycetous yeasts from soil on both islands, with species belonging to the Agaricomycotina found most often. Vishniac [9] reported that, when distribution of soil yeasts was studied on a latitude basis (the Antarctic, desert, the tundra and tropical forest) by canonical correspondence analysis, factors affecting distribution were: 42% associated with environmental variables, namely electrical conductivity, amount of rain, maximum growth temperature of the yeast concerned and the degree of oligotrophy; and the remaining 58% were undetermined. The distribution was also affected by the vegetation; *Cryptococcus albidus* and related species were found in desert soil, and species in the Cystofilobasidiales were common in grass. From this point of view, as the environments of both Iriomote and Rishiri Islands are treed areas, the result of this study that species in the Agaricomycotina were most frequently isolated was in accord with her report. Unfortunately, however, the number of common species common to the two sampling sites was quite small (only 15 species, see below), thus we are unable to discuss other probable factors affecting their distribution.

In this study, 60 and 46 new species candidates were anticipated from Iriomote Island and Rishiri Island, respectively. Although this result showed species diversity of Japan, this was obtained based on two sampling trips for each of the islands, indicating that species of the small population had probably not been isolated. The deep branches of ascomycetous yeasts (Figure S1 (a)) suggest a large number of taxa yet to be discovered. Furthermore, it was difficult to isolate yeast strains from soil samples of Iriomote Island, as filamentous fungi grew quickly and covered isolation plates even when they were incubated at 15°C. The slightly higher soil pH of Iriomote than that of Rishiri [10] may also have affected the number of yeasts present [11]. We think that it is likely that many undescribed species may be present at these sites.

### Comparison between Iriomote Island and Rishiri Island

The 15 common species on the two islands were: *Candida boleticola*, *Debaryomyces hansenii*, *Lipomyces lipofer* and *Wickerhamomyces anomalus* in Saccharomycotina, Ascomycota; *Bullera unica*, *Cryptococcus flavescens*, *C. laurentii*, *C. pseudolongus* and *Derxomyces boninensis*, in Agaricomycotina, *Sporobolomyces carnicolor*, *S. phaffii*, and *S. roseus* in Pucciniomycotina, Basidiomycota, and *Pseudozyma aphidis* in Ustilaginomycotina, Basidiomycota; and one new species candidate in each of Agaricomycotina and Pucciniomycotina, respectively. The remaining 168 species were isolated from a single location. Even for the commonly isolated species, the D1/D2 region of *D. hansenii*, *D. boninensis* and *S. roseus* showed different sequences (including in/dels) between the two islands. For *Sporobolomyces phaffii*, the isolation frequency was also quite different (Figure S1 (c)). These results suggest a heterogeneity in species diversity between the two areas.

From Iriomote Island, we isolated *Kazachstania yakushimaensis* and *Sporobolomyces ogasawarensis* which were also found on Yakushima in Kyushu and the Ogasawara Islands located around 1000 km south of Tokyo, respectively. *Rhodotorula bogoriensis* was also isolated from tropical areas. In addition, species which have been reported from other areas of Japan, European countries and USA, such as *Trichosporon porosum, Cryptococcus terricola* and *C. podzolicus* were isolated from Rishiri Island.

Small numbers of species common to both islands were also obtained in studies by other groups working with actinomycetes and filamentous fungi [10], [12], [13]. These reports showed similar tendencies of distribution, namely species found on Iriomote Island were similar to those of other subtropical areas, and those of Rishiri Island were similar to those of other cool temperate areas, suggesting that microbial inhabitants show their own marginal zone distinctiveness.

We divided the isolates into four groups for the analyses: IP, Iriomote-Plant; IS, Iriomote-Soil; RP, Rishiri-Plant; and RS, Rishiri-Soil. Based on sequences of the isolates, the diversity of the yeast community was compared among these groups using four methods: parsimony-test, unweighted UniFrac-test, weighted UniFrac-test and S-LIBSHUFF analysis, and each group was significantly distinct (p<0.001) by all methods. The results statistically supported the taxonomic diversity of the tentative identification.

### Richness of the Yeasts

Being aware that the number of OTUs at 0.5% difference of around 580 bp of the D1/D2 region of the rRNA gene (3-base difference) corresponds to the number of species [7], [8], the number of species in each group was estimated as shown in Table 2. In the plant samples, the chao1 estimated richness at a distance of 0.005 from Iriomote Island (153.1) was significantly higher than that from Rishiri Island (62.8). The rarefaction curves did not reach an asymptote, indicating that further sampling would have revealed additional diversity at this genetic resolution.

**Table 2:** The richness of yeasts isolated from Iriomote Island and Rishiri Island.

Distance	Source*	Chao	chao_lci	chao_hci	npshannon	shannoneven
unique	IP	187.230769	136.971731	294.935143	4.311526	0.892255
	IS	75	60.329788	115.507017	3.649379	0.863527
	RP	94.071429	75.594541	139.654933	3.331075	0.75049
	RS	94.333333	73.295956	151.363943	3.690856	0.850404
0.005	IP	153.142857	104.430918	277.349028	3.941275	0.872891
	IS	57.3	46.963289	89.164293	3.171349	0.786347
	RP	62.833333	52.409268	93.345356	2.924834	0.714072
	RS	66	54.335637	103.164103	3.425586	0.840266
0.03	IP	102.5	69.894665	206.48041	3.590733	0.863538
	IS	46.272727	40.346846	67.161694	3.018994	0.771407
	RP	36.5	33.032478	51.613017	2.381783	0.653084
	RS	57.5	45.118074	99.836961	3.189947	0.830502
0.1	IP	43	31.920488	96.112081	2.831796	0.822298
	IS	24.428571	23.193189	33.563816	2.44785	0.746375
	RP	28	24.807967	43.802788	1.662541	0.490269
	RS	43.166667	36.29218	70.658448	3.055918	0.838085

* IP, Iriomote-Plant; IS, Iriomote-Soil; RP, Rishiri-Plant; and RS, Rishiri-Soil.

**Figure 2:**
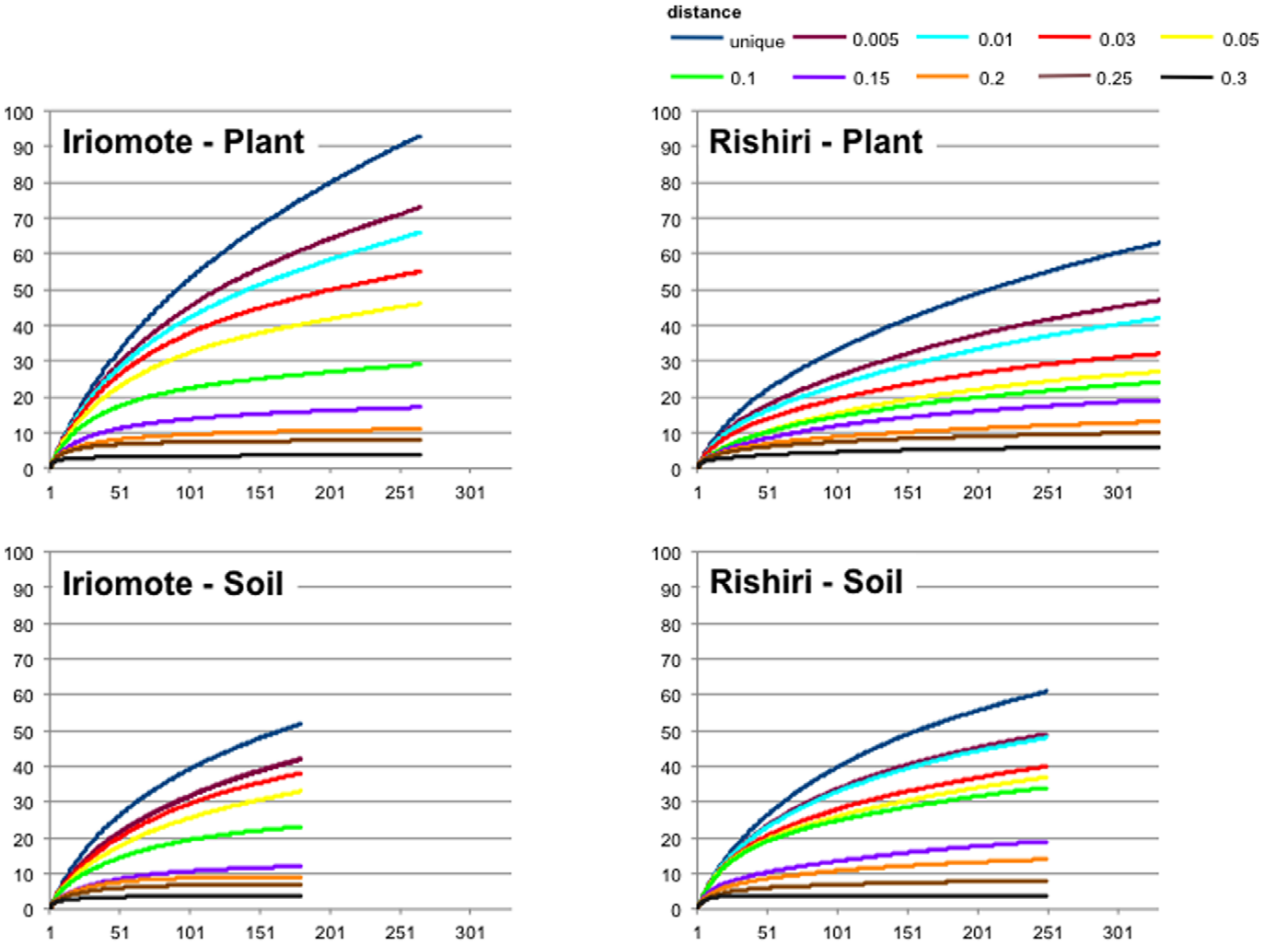
Comparison of rarefaction curves among plant and soil samples of Iriomote and Rishiri Islands. Rarefaction values were obtained using the MOTHUR program [25] and data of some particular thresholds of a respective group are plotted. Numerals in the boxes indicate the value of chao (chao_lci – chao_hci) of distances unique, 0.005, 0.03 and 0.01 from the top in the respective group.

The rarefaction curves of a respective group drawn based on some particular thresholds (Figure 2) showed that: the OTUs of both plants and soil from Iriomote and that of Rishiri plants decreased rather uniformly from distance 0.01 to 0.1, whereas the curves at distances 0.03 and 0.05 of Rishiri soil were similar. This suggested that, at a higher taxonomic level, the yeasts in Rishiri soils were more diverse than those of other samples. The richness of Rishiri soil at 0.1 thus became higher than that of Iriomote plants, although the number of unique OTU of Iriomote plant samples exceeded that of Rishiri soil samples. This indicates that not only species diversity but also a broader threshold level discussion may be valuable to understand the environment involved.

Although 183 species were detected from our samples, it is difficult to estimate how many genera or higher taxonomic levels are present when using the present taxonomic system of yeasts, as discussed. In addition, one third of new species candidates showed less than 97% similarity to described species as stated above; in such cases, it is difficult to find close relatives. To understand the microbial biodiversity in various environments, the study of richness and evenness of species level is fundamental, and that of the genus or higher level is also important and indispensable. In the ecological study of prokaryotes, the threshold of 95% similarity of 16S rRNA gene, which was introduced by Ludwig et al. [14], has been employed to estimate the richness at genus level; however, no such indicator has yet been introduced for the fungi.

Thus, to refer to each respective threshold value, we considered the report of Kurtzman [15], which reclassified the *Saccharomycetaceae* species by multigene analyses. Whereas Kurtzman showed the number of base differences of concatenated sequences between the genera in the paper, we counted the base differences of only the D1/D2 region of the LSU rRNA gene. The sequence between *S. cerevisiae* and *S. pastorianus* is around 2%, and those of *S. cerevisiae* and species in other genera is more than 3%; thus each OTU at a distance of 3% is assumed to be a well-defined group such as the genus *Saccharomyces*. When we compared the sequence similarities among respective genera, 40–50 base differences (approximately 8–10%) were most frequently detected (47%) (Table S1.1). A similar value was obtained for the basidiomycetous genera *Derxomyces, Dioszegia, Filobasidiella* and *Hannaella* that were reclassified according to phylogenetic relationships [16], [17] (Table S1.2), whereas a slightly larger value was obtained when the estimates of sequence divergence among yeast taxa in the Microbotryomycetes of the Pucciniomycotina was calculated according to the grouping shown in Fig. 100.7C of “The Yeasts, A Taxonomic Study” 5th ed. [17]. (Table S1.3) On the assumption that “distance 0.1″ and “0.03″ can be defined as indicators of the average range of genus level and that covering *Saccharomyces* sensu stricto, respectively, the taxonomic richness obtained in this study can be estimated as shown in Table 2.

The estimated richness at distances of 0.005, 0.03 and 0.1 from plant samples of Iriomote Island was significantly higher than that from Rishiri Island. In contrast, there were no significant differences in richness of soil samples between the two islands at distances of 0.005 and 0.03, the soil richness of the latter was significantly higher than that of the former at a distance of 0.1. Shannon diversity indices also supported this finding. In addition, the taxonomic diversity in a respective group was shown to be unique even when the diversity threshold range became broader, as shared richness at distances of 0.005, 0.03 and 0.1 showed similar patterns (Table S2).

### The Importance of Taxonomy in the Study of Biodiversity

The number of species included in “The Yeasts, A Taxonomic Study” 5^th^ ed. [1] increased some 1.8-fold over that of the 4^th^ ed. [18]. Reclassification based on phylogenetic relationships is particularly advanced for the ascomycetous teleomorphic genera, and the number of genera has increased from 45 to 73. However, no such comprehensive reclassification has yet been performed for the basidiomycetous yeasts, and the increase in number of genera was due to the finding of new genera based on new isolates. As a result, for the basidiomycetous yeasts, some teleomorphic genera were intermingled with a few anamorphic genera. Furthermore, since the morphological characteristics of yeasts (a phenotypic characteristic) are less distinct than those of filamentous fungi, such as mode of conidiogenesis, which is used to delineate species level in the filamentous fungi, morphology represents a poor choice for recognizing yeast genera. For example, delineation of anamorphic genera has been made by morphological characteristics such as *Bullera* and *Cryptococcus,* which were separated on the ability or inability to produce ballistoconidia.

A large number of sequences have been published using clone library methods for the analyses of fungal diversity in addition to sequences from isolated cultures. For example, filamentous fungi such as *Fusarium* (ascomycete) were most frequently detected in soil samples, and *Cryptococcus* species (basidiomycete) were the primary yeast species detected [19]. The name “*Cryptococcus”* used in the preceding paper was assumed to be a taxon belonging to the Agaricomycotina, but the genus *Cryptococcus* is a polyphyletic group and species in the genus belong to five orders: Cystofilobasidiales, Filobasidiales, Holtermaniellales, Tremellales and Trichosporonales [20]. Recently, some genera have been proposed for phylogenetically well-defined groups (for example, [17]), but the fundamental problem cannot yet be resolved, since the ill-defined species groups may remain in the genera *Bullera* or *Cryptococcus*. This indicates that it is difficult to identify an isolate to the genus level for basidiomycetous yeast species even with sequence data (see Figure S1 (a)–(d)).

A comprehensive reclassification of yeasts is necessary to understand the taxonomic richness in natural communities. The isolates reported in this study will be useful for such a reclassification in the future because they contain many new and taxonomically diverse yeast species. Moreover, using “distance 0.03” and “distance 0.1” as an indicator, “range of *Saccharomyces* sensu stricto” and “average range of genus level” would provide a better solution, although “distance 0.03” and “0.1” are only indicators to estimate the range of a phylogenetic group that corresponds to a particular size since the taxonomic delineation of a genus does not necessarily correspond to this threshold. The use of such an indicator, however, is tentatively proposed until reclassification of all yeasts is performed using phylogenetic analysis of multiple gene sequences.

## Materials and Methods

### Study Sites and Experimental Manipulation

Iriomote Island in the Iriomote-Ishigaki National Park and Rishiri Island in the Rishiri-Rebun-Sarobetsu National Park were selected as the sampling sites from which the microbiota were compared between the southern (subtropical zone) and northern (cool temperate zone) parts of the country. Samplings at both sites were carried out with the permission of the Ministry of Environment. On Iriomote Island in 2007, the lowest and highest natural air temperature was 11.5°C (January) and 34.8°C (July), respectively, and mean air temperature was 18.9°C (January) to 29.7°C (July); on Rishiri Island, they were −13.9°C (January) and 28.3°C (August), and the mean was −3.9°C (January) to 28.3°C (August). These temperature measurements were based on data of the Japan Meteorological Agency <http://www.data.jma.go.jp/obd/stats/etrn/index.php>. The vegetation of Iriomote Island is subtropical forest with a mangrove area, and that of Rishiri Island is conifer. Soil sample characteristics of Iriomote Island were yellow soil with mean pH 6.6 and an organic matter content of 10%; those of Rishiri Island were dark brown colored volcanic ash soil with mean pH 5.4 and an organic matter content of 18% [10].

Soil and plant samples were collected from Rishiri in August 2007 and September 2008, and from Iriomote in October 2007 and November 2008, respectively.

The isolation procedure was as follows: 0.5 g of soil sample was suspended in 5 ml of M3C [21] broth or sterilized distilled water after removing woody debris and roots, and was serially diluted (3, 9, and 27 fold); 0.1 ml of these dilutions was spread on media M3C [21] and DRBC Agar (BD-Difco) plates containing 50 µg/ml chloramphenicol (Wako, Osaka, Japan) and incubated at 15 or 20°C. Plant samples (leaves and fruits) were cut into small pieces and suspended, and 0.1 ml of suspension was used for the isolation. For plant samples, the ballistospore-fall method [22] was also employed. Colonies grown on the plates were selected under a stereomicroscope when their morphology seemed to be different from others on the same plate. Isolation work was continued for a maximum of one month unless filamentous fungi grew to cover the plate. To purify strains, selected colonies were re-streaked three times on fresh YM agar (BD-Difco) plates, and the procedure repeated three times. Isolates were kept at −80°C in YM broth supplemented with 10% glycerol before analyses.

### Sequencing and Tentative Identification of Isolates

Cells were suspended in 60 µl of Prepman Ultra Sample Preparation Reagent (Applied Biosystems) and template DNA for PCR was prepared according to the manufacturer’s instructions. The D1/D2 domain of LSU rRNA gene was amplified following Kurtzman & Robnett [23]. The PCR products were directly sequenced using an ABI Prism BigDye Terminator Cycle Sequencing Ready Reaction kit (Applied Biosystems) and analyzed with an Applied Biosystems sequencer model 3100 according to the manufacturer’s instructions. The sequences of D1/D2 domain of LSU rRNA gene used in this study are available from the DDBJ/GenBank/EMBL database (AB726265 – AB727285).

Tentative identification of isolates was performed using the megablast search of DDBJ/GenBank/EMBL. The sequence of isolates was compared with the type strain of closely related species; less than 3 base differences were treated as the same species [7], [8]. When an isolate was identified as belonging to a teleomorphic species, we used that teleomorphic name, although the identification was made only based on the sequence similarity; morphological, physiological or biochemical characteristics were not determined. In this study, strains in Pezizomycotina, such as *Aureobasidium pullulans*, were not included.

Representative strains have been deposited in the Japan Collection of Microorganisms (JCM) in RIKEN BioResource Center (JCM 16282–16305, 24501–24700).

### Phylogenetic Analyses, and Operational Taxonomic Unit Diversity and Composition Analyses

The sequences were aligned using the *MEGA* version 5 [24] and adjusted manually. A neighbor-joining tree was constructed using the *MEGA* version 5. A Kimura-2-parameter model was used for the substitution model, gaps were completely deleted, and bootstrap values were obtained from 1000 replications. Species richness and evenness (including parsimony test, unweighted and weighted UniFrac-test, and S-LIBSHUFF analysis) were determined using the MOTHUR program [25]. Since in/dels were usually counted for the species identification in yeasts, the “eachgap” option was used when the distant matrix of OTUs was calculated.

## Supporting Information

Figure S1 **A neighbor-joining tree of isolates based on the D1/2 region unique sequences.** (a) Saccharomycotina, (b) Ustilaginomycotina, (c) Pucciniomycotina and (d) Agaricomycotina. The evolutionary distance (refer to the bar) was calculated according to Kimura [26]. Numerals represent the percentages from 1000 replicating bootstrap samplings (a frequency of less than 60% is not shown) [27]. Reference taxa shown are closest relatives of the isolates retrieved from the DDBJ/GenBank/EMBL databases. The superscript “T” on the strain number indicates that the strain is the type strain of the species. Blue solid ring, new species candidate isolated from a plant of Iriomote Island; blue open ring, described species isolated from a plant of Iriomote; blue solid square, new species candidate isolated from soil of Iriomote; blue open square, described species isolated from soil of Iriomote; blue solid ring, new species candidate isolated from a plant of Rishiri; red open ring, described species isolated from a plant of Rishiri; red solid square, new species candidate isolated from soil of Rishiri; red open square, described species isolated from soil of Rishiri. Numerals in brackets after ring or square indicate the number of isolates. Asterisk after the brackets indicate that no close relatives (less than 97% sequence similarity) were found from the database. The hash mark after the brackets indicates that the sequence data are the same as that of type strain of the species. Yellow color on OTUs indicates this species was isolated from both Iriomote and Rishiri Islands.
(TIF)

Table S1 S1.1 Estimates of sequence divergence between genera in *Saccharomycetaceae*. Data were calculated based on Kurtzman [15]. ^a^Number of base differences per sequence averaging overall sequence pairs within each group. ^b^Upper right, Number of base differences per sequence averaging overall sequence pairs between groups; Lower left, The p-distance averaging overall sequence pairs between groups. All positions containing gaps and missing data were eliminated. There were a total of 515 positions in the final dataset. Evolutionary analyses were conducted in MEGA5 [24]. **S1.2 Estimates of sequence divergence between genera** ***Derxomyces*** **,** ***Dioszegia*** **,** ***Filobasidiella*** **and** ***Hannaella*** **.** Data were calculated based on Fig 100.8C of “The Yeasts, A Taxonomic Study” 5th ed. [17]. ^a^Number of base differences per sequence averaging overall sequence pairs within each group. ^b^Upper right, Number of base differences per sequence averaging overall sequence pairs between groups; Lower left, The p-distance averaging overall sequence pairs between groups. All positions containing gaps and missing data were eliminated. There were a total of 583 positions in the final dataset. Evolutionary analyses were conducted in MEGA5 [24]. **S1.3 Estimates of sequence divergence between yeast groups in the Microbotryomycetes of the Pucciniomycotina.** Data were calculated based on Fig 100.7C of “The Yeasts, A Taxonomic Study” 5th ed. [17]. ^a^ Number of base differences per sequence averaging overall sequence pairs within each group. ^b^ Upper right, Number of base differences per sequence averaging overall sequence pairs between groups; Lower left, The p-distance averaging overall sequence pairs between groups. All positions containing gaps and missing data were eliminated. There were a total of 569 positions in the final dataset. Evolutionary analyses were conducted in MEGA5 [24]. The presence of n/c in the results denotes cases in which it was not possible to estimate evolutionary distances.
(PDF)

Table S2 **The shared chaos between groups isolated from Iriomote Island and Rishiri Island on particular thresholds.**
(PDF)
