# Supplementary figures and images for: Taxonomic Richness of Yeasts in Japan within Subtropical and Cool Temperate Areas
Source: PLoS One. 2012 Nov 30;7(11):e50784. doi: 10.1371/journal.pone.0050784 (PMC3511277; doi:10.1371/journal.pone.0050784)

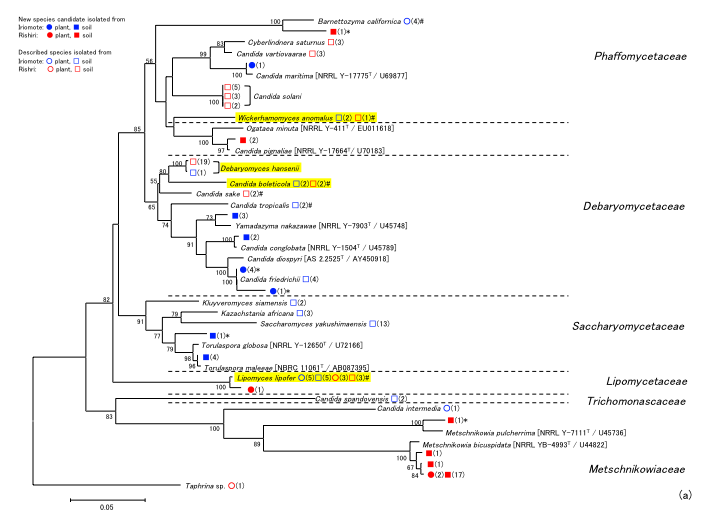

Supplement: Figure S1 — A neighbor-joining tree of isolates based on the D1/2 region unique sequences. (a) Saccharomycotina, (b) Ustilaginomycotina, (c) Pucciniomycotina and (d) Agaricomycotina. The evolutionary distance (refer to the bar) was calculated according to Kimura [26]. Numerals represent the percentages from 1000 replicating bootstrap samplings (a frequency of less than 60% is not shown) [27]. Reference taxa shown are closest relatives of the isolates retrieved from the DDBJ/GenBank/EMBL databases. The superscript “T” on the strain number indicates that the strain is the type strain of the species. Blue solid ring, new species candidate isolated from a plant of Iriomote Island; blue open ring, described species isolated from a plant of Iriomote; blue solid square, new species candidate isolated from soil of Iriomote; blue open square, described species isolated from soil of Iriomote; blue solid ring, new species candidate isolated from a plant of Rishiri; red open ring, described species isolated from a plant of Rishiri; red solid square, new species candidate isolated from soil of Rishiri; red open square, described species isolated from soil of Rishiri. Numerals in brackets after ring or square indicate the number of isolates. Asterisk after the brackets indicate that no close relatives (less than 97% sequence similarity) were found from the database. The hash mark after the brackets indicates that the sequence data are the same as that of type strain of the species. Yellow color on OTUs indicates this species was isolated from both Iriomote and Rishiri Islands. (TIF) [file pone.0050784.s001.tif]
