# Supplementary material for: Taxonomic Richness of Yeasts in Japan within Subtropical and Cool Temperate Areas
Source: PLoS One. 2012 Nov 30;7(11):e50784. doi: 10.1371/journal.pone.0050784 (PMC3511277; doi:10.1371/journal.pone.0050784)
Supplement: Table S1 — S1.1 Estimates of sequence divergence between genera in Saccharomycetaceae. Data were calculated based on Kurtzman [15]. aNumber of base differences per sequence averaging overall sequence pairs within each group. bUpper right, Number of base differences per sequence averaging overall sequence pairs between groups; Lower left, The p-distance averaging overall sequence pairs between groups. All positions containing gaps and missing data were eliminated. There were a total of 515 positions in the final dataset. Evolutionary analyses were conducted in MEGA5 [24]. S1.2 Estimates of sequence divergence between genera Derxomyces , Dioszegia , Filobasidiella and Hannaella . Data were calculated based on Fig 100.8C of “The Yeasts, A Taxonomic Study” 5th ed. [17]. aNumber of base differences per sequence averaging overall sequence pairs within each group. bUpper right, Number of base differences per sequence averaging overall sequence pairs between groups; Lower left, The p-distance averaging overall sequence pairs between groups. All positions containing gaps and missing data were eliminated. There were a total of 583 positions in the final dataset. Evolutionary analyses were conducted in MEGA5 [24]. S1.3 Estimates of sequence divergence between yeast groups in the Microbotryomycetes of the Pucciniomycotina. Data were calculated based on Fig 100.7C of “The Yeasts, A Taxonomic Study” 5th ed. [17]. a Number of base differences per sequence averaging overall sequence pairs within each group. b Upper right, Number of base differences per sequence averaging overall sequence pairs between groups; Lower left, The p-distance averaging overall sequence pairs between groups. All positions containing gaps and missing data were eliminated. There were a total of 569 positions in the final dataset. Evolutionary analyses were conducted in MEGA5 [24]. The presence of n/c in the results denotes cases in which it was not possible to estimate evolutionary distances. (PDF) [file pone.0050784.s002.pdf]

Table S1.1 Estimates of sequence divergence between genera in *Saccharomycetaceae*.

	No. of species included	Intrageneric nucleotide changes[a]		Intergeneric nucleotide changes[b]												
		No. of base differences	No. of base differences per site	*Kaz.*	*Nak.*	*Ere.*	*Han.*	*Zyg.*	*Z'tor.*	*Sac.*	*Tet.*	*Lac.*	*Tor.*	*Nau.*	*Klu.*	*Van.*
Kazachstania	19	29.44	0.064		47.46	55.93	50.20	40.95	50.58	27.80	66.98	39.21	30.27	28.21	47.83	34.00
Nakaseomyces	4	44.33	0.108	0.09		60.50	60.46	42.79	45.25	42.07	69.45	45.60	41.40	40.88	53.58	47.75
Eremothecium	5	17.80	0.042	0.11	0.12		59.54	52.93	62.00	49.26	76.00	39.72	46.48	44.20	47.53	47.50
Hanseniaspora	7	30.76	0.076	0.10	0.12	0.12		52.83	63.14	48.71	78.20	45.71	39.29	42.29	54.69	40.86
Zygosaccharyomyces	6	21.07	0.056	0.08	0.08	0.10	0.10		46.17	34.83	68.67	35.83	35.30	32.75	47.19	37.25
Zygotorulaspora	2	20.00	0.045	0.10	0.09	0.12	0.12	0.09		46.50	76.10	45.00	44.90	40.25	52.42	50.25
Saccharomyces	7	12.10	0.029	0.05	0.08	0.10	0.09	0.07	0.09		63.69	34.74	21.97	23.43	42.60	23.07
Tetrapisispora	5	48.30	0.108	0.13	0.13	0.15	0.15	0.13	0.15	0.12		68.04	62.76	61.30	71.77	69.20
Lachancea	5	16.50	0.033	0.08	0.09	0.08	0.09	0.07	0.09	0.07	0.13		28.36	27.10	37.33	27.50
Torulaspora	5	5.20	0.013	0.06	0.08	0.09	0.08	0.07	0.09	0.04	0.12	0.06		16.90	32.00	20.60
Naumovozyma	2	7.00	0.021	0.05	0.08	0.09	0.08	0.06	0.08	0.05	0.12	0.05	0.03		35.75	24.75
Kluyveromyces	6	9.20	0.022	0.09	0.10	0.09	0.11	0.09	0.10	0.08	0.14	0.07	0.06	0.07		45.08
Vanderwaltozyma	2	12.00	0.023	0.07	0.09	0.09	0.08	0.07	0.10	0.04	0.13	0.05	0.04	0.05	0.09	

Data were calculated based on Kurtzman [15].

[a] Number of base differences per sequence averaging overall sequence pairs within each group.

b Upper right, Number of base differences per sequence averaging overall sequence pairs between groups; Lower left, The p-distance averaging overall sequence pairs between groups.

All positions containing gaps and missing data were eliminated.
There were a total of 515 positions in the final dataset. Evolutionary analyses were conducted in MEGA5 [24].

Table S1.2 Estimates of sequence divergence between genera *Derxomyces, Dioszegia, Filobasidiella* and *Hannaella* .

	No. of species included	Intrageneric nucleotide changes[a]		Intergeneric nucleotide changes[b]			
		No. of base differences	No. of base differences per site	*Der.*	*Dio.*	*Fil.*	*Han.*
Derxomyces	11	13.58	0.024		49.60	75.59	46.05
Dioszegia	13	15.05	0.026	0.09		90.23	56.76
Filobasidiella	4	18.83	0.033	0.12	0.16		68.96
Hannaella	7	28.57	0.050	0.08	0.10	0.12	

Data were calculated based on Fig 100.8C of "The Yeasts, A Taxonomic Study" 5th ed. [17].

[a] Number of base differences per sequence averaging overall sequence pairs within each group.

[b] Upper right, Number of base differences per sequence averaging overall sequence pairs between groups; Lower left, The p-distance averaging overall sequence pairs between groups.

All positions containing gaps and missing data were eliminated. There were a total of 583 positions in the final dataset. Evolutionary analyses were conducted in MEGA5 [24].

Table S1.3 Estimates of sequence divergence between yeast groups in the Microbotryomycetes of the Pucciniomycotina.

	No. of species included	Intra-group nucleotide changes[a]		Inter-group nucleotide changes[b]												
		No. of base differences	No. of base differences per site	*yam. / L. ant.*	Leuco-spori-diales	Micro-botry-ales	*van.*	*Cur.*	*Col.*	*hyl.*	*H. pyc.*	*jav.*	*Rho.*	mixed *Rho. / Spo.*	*Spo.*	*son.*
yamatoana / Leucosporidium antarcticum	21	32.28	0.060		38.71	48.13	34.43	45.43	47.11	56.62	55.76	58.90	60.93	61.01	63.02	86.76
Leucosporidiales	8	10.71	0.020	0.07		33.02	21.63	41.00	55.79	65.38	64.00	69.00	59.68	52.79	71.45	91.75
Microbotryales	12	21.03	0.039	0.09	0.06		37.38	49.71	58.24	67.92	65.33	76.33	60.19	52.95	72.24	86.58
vanillica	2	5.00	0.009	0.06	0.04	0.07		31.25	51.75	60.00	58.50	58.50	56.58	52.39	66.32	87.50
Curvibasidium	4	11.50	0.021	0.08	0.08	0.09	0.06		55.75	63.25	67.75	65.75	62.50	58.97	68.93	85.25
Colacogloea	6	30.93	0.057	0.09	0.10	0.11	0.09	0.10		61.67	64.33	62.50	66.69	70.61	74.32	92.17
Rhodotorula hylophila	1	n/c		0.11	0.13	0.14	0.12	0.13	0.12		58.00	61.00	66.69	70.89	68.12	88.00
Heterogastridium pycnidioideum	1	n/c		0.11	0.12	0.12	0.11	0.13	0.12	0.12		68.00	81.38	84.00	79.76	101.00
Rhodotorula javanica	1	n/c		0.11	0.13	0.14	0.11	0.12	0.12	0.12	0.13		71.31	77.78	79.94	102.00
Rhodosporidium	13	24.74	0.047	0.12	0.11	0.11	0.11	0.12	0.13	0.13	0.15	0.13		40.78	66.71	90.15
mixed *Rhodosporidium / Sporidiobolus*	9	19.86	0.038	0.12	0.10	0.10	0.10	0.11	0.13	0.14	0.16	0.15	0.08		57.56	83.78
Sporidiobolus	17	20.75	0.039	0.12	0.14	0.14	0.13	0.13	0.14	0.14	0.15	0.15	0.13	0.11		83.53
Rhodotorula sonckii	1	n/c		0.16	0.17	0.16	0.16	0.16	0.17	0.18	0.19	0.19	0.17	0.16	0.16	

Data were calculated based on Fig 100.7C of "The Yeasts, A Taxonomic Study" 5th ed. [17].

[a] Number of base differences per sequence averaging overall sequence pairs within each group.

[b] Upper right, Number of base differences per sequence averaging overall sequence pairs between groups; Lower left, The p-distance averaging overall sequence pairs between groups.
All positions containing gaps and missing data were eliminated. There were a total of 569 positions in the final dataset. Evolutionary analyses were conducted in MEGA5 [24].
The presence of n/c in the results denotes cases in which it was not possible to estimate evolutionary distances.
