# Supplementary material for: Taxonomic Richness of Yeasts in Japan within Subtropical and Cool Temperate Areas
Source: PLoS One. 2012 Nov 30;7(11):e50784. doi: 10.1371/journal.pone.0050784 (PMC3511277; doi:10.1371/journal.pone.0050784)
Supplement: Table S2 — The shared chaos between groups isolated from Iriomote Island and Rishiri Island on particular thresholds. (PDF) [file pone.0050784.s003.pdf]

Table S2 The shared chaos between groups isolated from Iriomote Island and Rishiri Island on particular thresholds

Distance	IP	IS	RP	RS	Shared between										Total
					IP and IS	IP and RP	IP and RS	IS and RP	IS and RS	RP and RS	IP, IS and RP	IP, IS and RS	IP, RP and RS	IS, RP and RS	
unique	187.231	75	94.0714	94.3333	39.3333	10.5	5	2	10.1667	5.5	2	4	2	2	386.136
0.005	153.143	57.3	62.8333	66	37	17.5	6	2	11.1667	7.5	2	5	2	2	267.109
0.030	102.5	46.2727	36.5	57.5	31	20.5	6	4	9.41667	6	4	4	3	2	176.856
0.100	43	24.4286	28	43.1667	17.5	19.5	17.75	7.5	16	12.75	7.5	10.5	10.3333	7.5	75.9286
